# Acoustic fabrics enabled by piezoelectric polymer fibers

**DOI:** 10.1093/nsr/nwac098

**Published:** 2022-05-20

**Authors:** Xiaoming Tao

**Affiliations:** Research Institute for Intelligent Wearable Systems, Institute of Textiles and Clothing, Hong Kong Polytechnic University, China

Some 15 years ago, Prof. Yoel Fink at MIT inspired the world by proposing multimaterial fibers that can see, hear, sense and communicate [1] by integrating microelectronic devices into single thermally drawn fiber or fiber assembly. Since then, great progress has been made around the globe [2] to impart these functions into fabrics with such fiber-based devices. A large-area fabric was envisaged then to function as a robust and damage-tolerating acoustic

camera that is able to detect not only the amplitude but also the direction of the sound propagation; thus, a spatial map can be built on the fiber network. The recent paper [3] published in *Nature* by Wei Yan *et al.* is indeed part of the exciting progress being made by Prof. Fink's group in this direction. The three examples illustrate interesting potential applications: a woven shirt with dual acoustic fibers measuring the direction of an acoustic impulse, bidirectional communications established between sound-emitting and sound-receiving fabrics, and a shirt auscultating cardiac sound signals.

**Figure 1.**
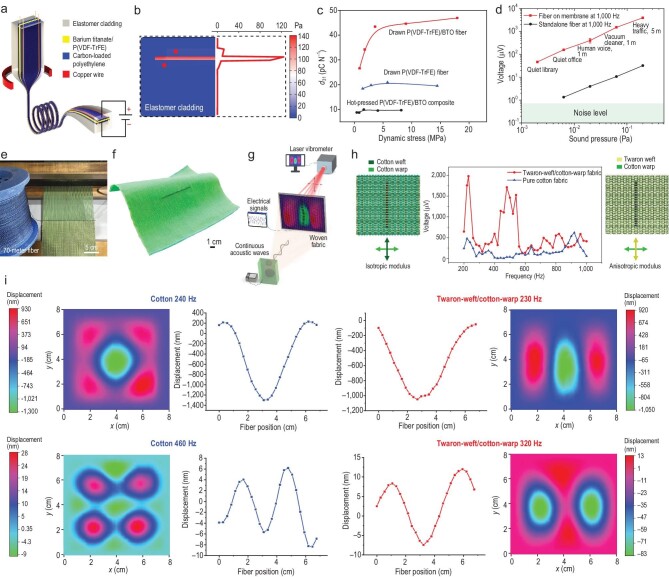
Piezoelectric fiber and fabric [3]. (a) Fiber fabrication and structure. (b) Stress distribution. (c) Fiber properties. (d) Output signal under various acoustic conditions. (e) The fiber with a length of ∼70 m is incorporated into the fabric during the weaving process. (f) A photograph of the acoustic fiber containing one fiber transducer (6.7 cm in length). (g) Acoustic measurement set-up. (h) Measured frequency response of the fabric sample (8 × 8 cm); the schematics of the anisotropic fabric (right) and the orthogonally isotropic fabric (left) are also shown. (i) Measured vibrational modes together with displacement perpendicular to the fabric surface of the two fabrics and fibers at their resonance and anti-resonance frequencies using the laser vibrometer. Copyright 2022, Springer Nature.

The piezoelectric fiber transducer is made of a P(VDF-TrFE)/BTO piezoelectric composite layer that is sandwiched between conducting carbon/polyethylene electrodes and is encapsulated in a softer poly(styrene-b-ethylene-co-butylene-b-styrene) (SEBS) cladding by the thermal drawing process, as shown in Fig. 1a. The off-central arrangement ensures a satisfactory sensitivity via the higher stress level in the piezoelectric layer during the off-plane bending (Fig. 1b). The drawn piezocomposite fiber has a high piezoelectric charge coefficient of ∼46 pC/N (Fig. 1c).

The electrical signals received from film devices mounted with a fiber transducer have been greatly enhanced as compared with those from a single fiber. The mechanical vibrations of the film have indeed magnified the sound-wave signals by two orders of magnitude (Fig. 1d). The theoretical analysis and simulation of a thin shell were based on the film device but sufficiently addressed

the essential and major features of more complex fabric devices.

The authors used two fabrics with orthogonally isotropic (cotton/cotton) and anisotropic (Twaron/cotton) structures and observed the low-order mechanical vibration modes converting from 10^−7^-atmosphere pressure waves at audible frequencies, as shown in Fig. 1e–h and i. Concurrent measurements of electric output and spatial vibration patterns in response to audible acoustic excitation reveal that fabric vibrational modes with nanometer amplitude displacement are the source of the electrical output of the fiber. The acoustic measurement of the samples was conducted by fixing them on an acoustic insulating frame with a given sample size (Fig. 1g). The numeric simulation was also using the same boundary conditions. However, free or partially constrained boundary conditions or device size often occur when a fabric is in use, which may change the vibration modes and thus the responses. The acoustic sensitivity is also frequency-dependent. Further studies on the magnification mechanism may shed light on the frequency-related relationship between the size, structure and response of the fabric with the piezoelectric fibers.

The signal strength from the fiber may be further improved by replacing the two-phase co-polymer with a new four-phase co-polymer (P(VDF-TrEE-CFE-FA))

[4], which, in a low electric field, exhibits ultra-high electromechanical coupling constant and piezoelectric coefficient comparable or even better than the inorganic piezoelectric ceramic (ZrTi)O_3_. Other dielectric polymer materials capable of mechanoelectrical energy conversion may offer alternative ways for acoustic fibers and fabrics such as triboelectric and electret materials. Compared with piezoelectric fibers, these alternative materials may face the stability challenge of a humidity effect. In view of that, I am certain that more follow-up work will reveal more opportunities to promote further development of acoustic fabrics.

***Conflict of interest statement***. None declared.
